# Terpinen-4-ol Improves the Intestinal Barrier Function of the Colon in Immune-Stressed Weaning Piglets

**DOI:** 10.3390/ani15010009

**Published:** 2024-12-24

**Authors:** Lihuai Yu, Guangzhi Qiu, Xiaomu Yu, Jianwei Zhao, Jun Liu, Hongrong Wang, Li Dong

**Affiliations:** 1 College of Animal Science and Technology, Yangzhou University, Yangzhou 215009, China; lhyu@yzu.edu.cn (L.Y.); xiaonanhaiqiu@163.com (G.Q.); 19551655575@163.com (X.Y.); zhaojianwei@anschina.cn (J.Z.); shiqin12141997@163.com (J.L.); hrwang@yzu.edu.cn (H.W.); 2 Institute of Animal Nutrition, Sichuan Agricultural University, Chengdu 611130, China

**Keywords:** terpinen-4-ol, weaned piglets, colon, antioxidant function, immune function, microbiome

## Abstract

The aim of this study was to investigate the effects of terpinen-4-ol (TER) supplementation on the intestinal barrier function of pigs. Five groups of fifty 28-day-old piglets with comparable body weights were randomly assigned to the following groups: the control group (CON), the lipopolysaccharide group (LPS), the low TER group (PLT), the middle TER group (PMT), and the high TER group (PHT). The basal diet was given to the CON and LPS groups, and 30, 60, or 90 mg/kg TER was added to the basal diet for the TER groups. After the 21-day trial period, piglets in the LPS and TER groups received an intraperitoneal injection of 100 μg/kg body weight of LPS, whereas the piglets in the CON group received an injection of 0.9% normal saline solution. The results showed that LPS stimulation resulted in a decrease (*p* < 0.05) in the depth of colonic crypts in piglets, which was greater (*p* < 0.05) in the TER group. Compared with those in the CON group, the number of goblet cells and MUC2 expression were decreased in the colon of piglets in the LPS group, while these parameters were increased in the PMT group (*p* < 0.05). The malondialdehyde (MDA) content was greater in the colon of the LPS group than in that of the CON group, while the activities of glutathione peroxidase (*GSH-Px*), superoxide dismutase (*SOD*), and catalase (*CAT*) were lower in the colon of the LPS group; conversely, the MDA content was lower in the colons of the PLT and PMT groups than in those of the LPS group (*p* < 0.05). TER also reduced (*p* < 0.05) LPS-induced upregulation of *IL-1β* and *TNF-α* expression, along with the relative gene expression of *NLRP3*, *ASC*, and *caspase-1* in the colon of piglets (*p* < 0.05). Compared with those in the CON group, the abundances of *Firmicutes* and *UCG-005* in the LPS group were lower (*p* < 0.05), and those in the TER group were significantly greater than those in the LPS group. Compared with those in the CON group, the abundance of *Proteobacteria* in the LPS group increased (*p* < 0.05), while the abundance of *Actinobacteria* and *Phascolarctobacterium* increased (*p* < 0.05) in the colon of the PHT group compared with that in the LPS group. In conclusion, TER effectively improved the intestinal barrier function of the colon in weaning piglets. Based on the results of this study, the appropriate dose of TER in the diets of weaning piglets was 60 mg/kg.

## Simple Summary

The implications of intestinal barrier function improvement in immune-stressed weaning piglets by terpinen-4-ol(TER) are observed. Enhanced nutrient absorption, reduced gastrointestinal disease incidence, and improved overall growth performance can be achieved through improved intestinal barrier function. A promising alternative is offered by the use of TER as a natural plant extract. Far-reaching implications on animal health, economic viability, environmental sustainability, and animal welfare are observed from the use of TER to improve intestinal barrier function in immune-stressed weaning piglets. The importance of natural alternatives in enhancing livestock productivity and health is underscored by this promising approach, paving the way for more sustainable and ethical farming practices.

## 1. Introduction

Once pathogenic microbes such as *Salmonella* and *Escherichia coli* are present in the raising environment, they might stimulate the piglets’ immune systems, leading to inflammatory reactions and a greater frequency of diarrhea [1], which can hinder the growth of piglets and result in subsequent economic losses. Consequently, preserving the integrity and functionality of the intestines is essential for animal health [2]. Inflammatory bowel disease (IBD) is a chronic gastrointestinal inflammatory condition characterized primarily by weight loss and diarrhea, which can be induced by dioctyl sodium sulfosuccinate (DSS) stimulation [3]. Although DSS and LPS trigger inflammatory responses through different initial mechanisms, they are linked in some ways. DSS and LPS stimulation have similar inflammatory mediators including *TNF-α* and *IL-1β* [4]. In addition, DSS and LPS stimulation can both cause a leaky gut effect. DSS may damage the integrity of the intestinal mucosal barrier, thus causing the invasion of the components of the bacteria, including LPS, thus indirectly triggering systemic inflammatory responses similar to those caused by LPS [5]. IBD involves lesions in the colonic mucosa and submucosa, leading to the loss of intestinal barrier integrity [6]. Previous research has indicated that colon inflammation can be induced by LPS stimulation, resulting in weight loss and diarrhea in mice [7]. The primary ingredient in tea tree oil is TER, which has antibacterial, anti-inflammatory, and other properties [8]. Additionally, our previous study demonstrated that tea tree oil, a plant extract whose main component is TER, can alleviate the inflammatory damage to the small intestine caused by weaning, thereby improving growth performance and reducing the frequency of diarrhea in piglets. Therefore, LPS was hypothesized to cause colonic inflammatory damage in piglets and that the addition of TER may alleviate colonic inflammatory damage.

Intestinal barriers mainly include physical barriers, chemical barriers, microbial barriers, and immune barriers [9], which interact with each other to maintain intestinal health. Close intercellular connections and intestinal epithelial cells make up most of the physical barriers [10]. Digestive enzymes and MUC2 are two examples of the substances that make up the chemical barriers [11]. The primary components of the immunological barriers are cytokines (*IL-1β*, *TNF-α*, *IL-10*, and *IL-18*) and antibodies (sIgA) released by intestinal plasma cells [12]. An important component of the microbial barrier is the intestinal flora; the intestinal flora maintains the stability of the intestinal microecosystem [13]. A previous study suggested that DSS stimulation severely impaired morphology, decreased crypt depth, decreased the number of goblet cells, and downregulated MUC2 expression, while TER (a terpenoid) alleviated mucosal integrity and injury to the colon in mice [14]. Another study also showed that DSS led to decreased *SOD* activity and increased MDA levels, while ursolic acid (UA) (another terpenoid) alleviated these changes in antioxidant enzymes in the colon of mice [15]. Previous research has shown that, following LPS stimulation, the colons of mice exhibit a large decrease in the levels of anti-inflammatory cytokines such as *IL-10* and a dramatic increase in the levels of proinflammatory cytokines such as *IL-1β*, *IL-18*, and *TNF-α* [16]. Moreover, the addition of terpenoid-containing Lonicerae japonicae flos may help offset the increase in proinflammatory cytokine synthesis and the decrease in anti-inflammatory cytokine release [17]. Hence, it was hypothesized that, by controlling the intestinal barrier function of the colon, TER may reduce the risk of diarrhea and enhance the ability of piglets to thrive. The intestinal morphology, goblet cell counts, MUC2 expression, antioxidant enzyme activity, cytokine production, and intestinal microbial population in the colon of immune-stressed weaned pigs were used in this investigation to assess the TER regulation of the intestinal barrier function. This study was the first to investigate the colonic function of immune-stressed weaned piglets. The results of this study may provide a new reference for the application of TER as a new type of feed additive and for the nutritional regulation of intestinal health in both piglets and humans with colitis.

## 2. Materials and Methods

### 2.1. Ethical Statement

All procedures of this experiment were carried out in accordance with the protocol approved by the Experimental Animal Ethics Committee of Yangzhou University (approval number: 202302040).

### 2.2. Animals and Experimental Design

Fifty 28-day-old piglets (weaned at 21 days) with comparable body weights (Duroc × Landrace × Yorkshire, 7.5–8 kg) were divided into five groups at random, with half of the groups being female and the other half being male: the LPS group, the low-, intermediate-, and high-dose TER groups, and the CON group. The CON and LPS groups were fed a basal diet, while the TER group was fed a basal diet supplemented with 30, 60, or 90 mg/kg TER. At the end of the 21-day trial period, weaned piglets in the LPS- and TER-supplemented groups were intraperitoneally injected with LPS at a dose of 100 µg/kg body weight, whereas the control group received an injection of normal saline solution (0.9%). Six piglets from each group were randomly selected (n = 6). Samples were taken 6 h after LPS or saline stimulation.

This study was carried out at the piglet test site of Yiluxian Agricultural Technology Limited Company in Shaobo Town, Yangzhou City, Jiangsu Province, China. Every experimental piglet was reared in a single building. The room was maintained at 25–30 °C and approximately 65% relative humidity. The piglets were fed four times a day at 7:00, 11:00, 15:00, and 19:00, and they had unlimited access to food and water. The experimental diets of piglets in this study referred to the need of piglets in the Chinese National Standard of the Nutrient Requirement of Swine (GB/T 39235-2020). The feed formula and nutritional value are shown in Table 1.

### 2.3. Sample Collection

After being stimulated with LPS for six hours, six piglets from each group were randomly chosen and were sacrificed. The colon was removed from the weaned piglets by opening their abdomens. The chyme was gently rinsed off with PBS, and the intestinal segment was longitudinally opened. Colon tissue samples were collected into 2 mL cryovials and stored in a −80 °C freezer for subsequent determination of cytokine levels and gene expression. In addition, the intestinal segments were cut, immersed in 4% paraformaldehyde, and then stored at 4 °C for subsequent histological examination.

### 2.4. Histological Study

Formaldehyde (4%) was used to treat the colon. Following dehydration, sectioning, hematoxylin and eosin staining, and paraffin embedding were performed. As previously mentioned, tissue slices were examined and imaged with a camera using optical microscopy (Olympus IX53, Olympus Optical Co., Ltd., Tokyo, Japan) [18].

### 2.5. Goblet Cell Number Analysis

To examine the goblet cells, a periodic acid–Schiff (PAS) staining method targeting polysaccharides was applied utilizing a commercially available kit from Solarbio Science & Technology Co., Ltd. (Beijing, China). Observations were made at a magnification of 200× with an Olympus microscope, and data were captured via a Nikon H550L digital camera across ten distinct viewing fields. In each of the 15 separate intestinal crypts, 100 enterocytes were analyzed for the presence of goblet cells [19].

### 2.6. Immunohistochemical Analysis

As previously described, immunohistochemistry analysis was used to determine the location of MUC2 proteins in the colon [20]. The tissue sections were treated with antibodies specific for the MUC2 protein, which were obtained from Proteintech (Rosemont, IL, USA). The primary MUC2 antibody was prepared at a dilution of 1:1500 and allowed to incubate overnight at 4 °C. Using a 200× magnification Olympus fluorescein microscope with DP2-BSW software, digital pictures were taken. For image analysis and processing, ImageProline Plus 5.1 (Media Cybernetics, Rockville, MD, USA) was utilized. The relative MUC2 abundance was quantified by calculating the ratio of the integrated optical density to the area.

### 2.7. Determination of the Levels of Antioxidant Indicators

Superoxide dismutase (*SOD*; No: BC5165), malondialdehyde (MDA, No: BC0025), catalase (*CAT*, No: BC0205), glutathione peroxidase (*GSH-Px*, No: BC1195), and total antioxidant capacity (T-AOC, No: BC1315) were among the antioxidant enzymes whose activity was measured in accordance with the manufacturer’s guidelines. All the kits used in the experiment were purchased from Solarbio Science & Technology Co., Ltd., Beijing, China.

### 2.8. Cytokine Level Analysis by ELISA

A total of 0.1 g of intestinal mucosa sample was homogenized in an ice box after being combined with 900 μL PBS. Afterwards, centrifugation was carried out for ten minutes at 4 °C at 6000× *g*. The supernatant was collected, and the levels of *IL-1β* (Solebao, SEKP-0001), *IL-10* (Solebao, MM-042301), and *TNF-α* (Solebao, SEKP-0009) were determined according to the manufacturer’s instructions. All the kits used in the experiment were purchased from Solarbio Science & Technology Co., Ltd., Beijing, China.

### 2.9. mRNA Expression Analysis by Real-Time PCR

In the colonic mucosa, the gene expression levels of *CAT*, *SOD1*, *GPX1*, *NLRP3*, *ASC*, *caspase-1*, *IL-1β*, *IL-18*, and *TNF-α* were quantified. According to the manufacturer’s instructions, frozen colon tissue was subjected to RNA extraction using TRIzol reagent. The quality of the RNA was assessed via agarose gel electrophoresis. According to the manufacturer’s instructions, a reverse transcription kit was used to produce complementary cDNA from RNA. (Vazyme, R323.01). Primers were designed through an NCBI PubMed query and synthesized by Shenggong Bioengineering Co., Ltd., Shanghai, China. The primer sequences are provided in Table 2. Using the β-actin gene as an internal reference, the 2^−△△CT^ technique was used to quantify the relative expression of the target gene [21]. All reverse transcription and quantitative fluorescence reagents used in this study were purchased from Novozen Biotechnology Co., Ltd., Nanjing, China.

### 2.10. Analysis of the 16S rDNA Microbiota in the Colon Contents

Whole bacterial DNA from the feces of the gut microbiota was extracted using the CTAB technique. The forward primer 341 F (5′-CCTACGGGNGGCWGCAG-3′) and reverse primer 805 R (5′-GACTACHVGGGTATCTAATCC-3′) were used to amplify the bacterial 16S rRNA gene (V3–V4). Hangzhou Lianchuan Biotechnology Co., Ltd. (Hangzhou, China) used the Illumina MiSeq platform and the MiSeq Reagent Kit V3 to purify, quantify, pool, and sequence the amplicons. The QIIME program (v1.8.0) was used to examine the data [22].

### 2.11. Statistical Analysis

The data were analyzed using Excel 2021 software and SPSS 19.0 software, and multiple comparisons were made between the groups using Duncan’s test or one-way ANOVA. *p* < 0.05 represented significant differences, and *p* < 0.01 represented extremely significant differences.

## 3. Results

*Study Number of goblet cells and expression of MUC2*

**Figure 1:**
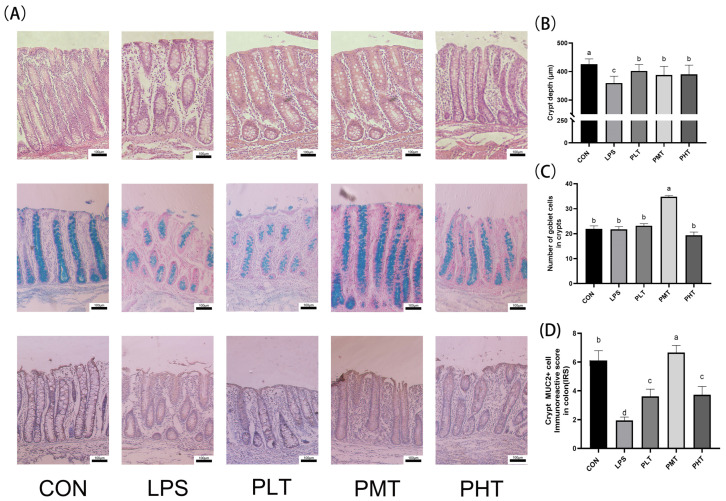
The effect of terpinen-4-ol on the colon morphology and structure of immune-stressed piglets. (**A**) Colon tissue HE, PAS, and immunohistochemical staining (100× magnification). (**B**) Depth of crypt. (**C**) Number of goblet cells. (**D**) MUC2 positive area. The data are represented as mean ± SD, n = 8. Different letters indicate significant differences (*p* < 0.05).

The effects of TER on the morphology and structure of the colon in immune-stressed piglets are shown in Figure 1. Compared with that in the CON group, the crypt depth in the colon of piglets in the LPS group was significantly lower (*p* < 0.05). Compared with piglets in the LPS group, piglets in the TER group had more complete colonic crypts, less damage, and a significant increase in crypt depth (*p* < 0.05) (Figure 1B). However, the number of goblet cells in the PMT group was significantly greater than that in the CON group and the LPS group (*p* < 0.05) (Figure 1C). The immunohistochemistry results showed that the expression of the MUC2 protein in the colon of weaned piglets in the LPS group was significantly lower (*p* < 0.05) than that in the colon of weaned piglets in the CON group, while the expression of the MUC2 protein in the colon was significantly greater (*p* < 0.05) in the TER-supplemented group than in the LPS group (Figure 1D).

*Gene expression and antioxidant enzyme activity*

**Figure 2:**
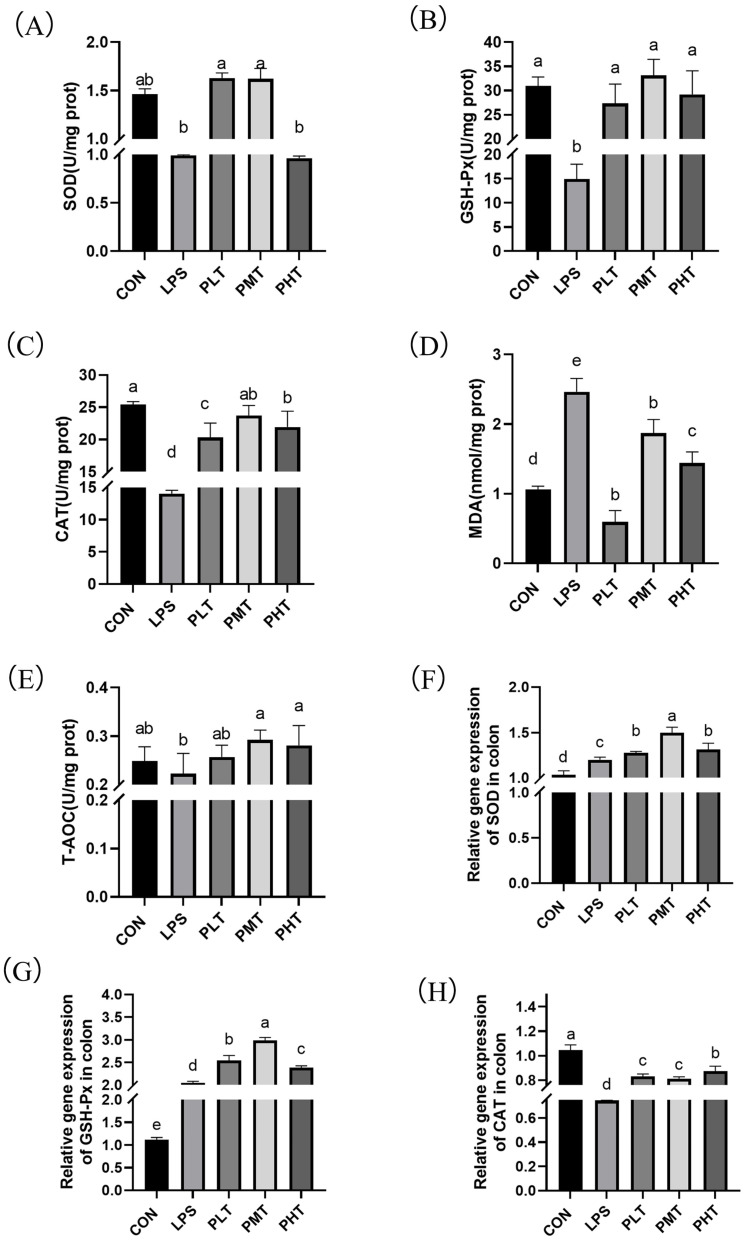
The effect of terpinen-4-ol on the activity and gene expression of antioxidant enzymes in the colon of immune-stressed piglets. (**A**) *SOD* enzyme activity. (**B**) *GSH-Px* enzyme activity. (**C**) *CAT* enzyme activity. (**D**) MDA enzyme activity. (**E**) T-AOC enzyme activity. (**F**) *SOD* gene expression. (**G**) *GSH-Px* gene expression. (**H**) *CAT* gene expression. The data are represented as mean ± SD, n = 8. Different letters indicate significant differences (*p* < 0.05).

The effects of TER on antioxidant enzyme activity and gene expression in the colon of immune-stressed piglets are shown in Figure 2. The piglets in the LPS group had greater MDA content in their colons (*p* < 0.05) than those in the CON group. (Figure 2D), while the activities of *GSH-Px*, *SOD*, and *CAT* decreased (*p* < 0.05). The MDA concentration in the colons of the PLT and PMT groups was lower than that in the colons of the LPS group (*p* < 0.05), while the activities of *GSH-Px*, *CAT*, and *SOD* increased (*p* < 0.05) (Figure 2A–C,E). Moreover, the gene expression of these antioxidant enzymes was also measured. In the TER group, there was a significant increase (*p* < 0.05) in the expression of the *GPX1* and *SOD* genes compared with that in the CON group; however, the expression of the *CAT* gene decreased. *CAT*, *GPX1*, and *SOD* gene expression in the TER group was greater (*p* < 0.05) than that in the LPS group (Figure 2F–H).

*Cytokine content and gene expression*

**Figure 3:**
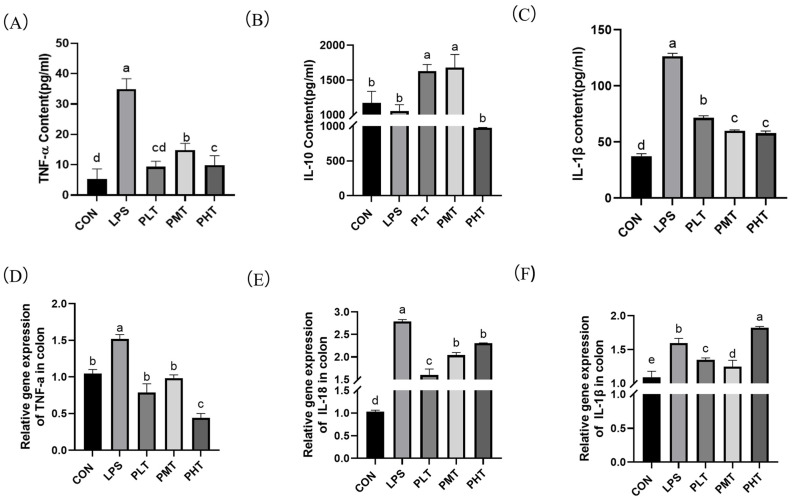
The effect of terpinen-4-ol on the content and gene expression of colitis factors in immune-stressed piglets. (**A**) *TNF-a* content. (**B**) *IL-1β* content. (**C**) *IL-10* content. (**D**) *TNF-a* gene expression and (**E**) *IL-18* gene expression. (**F**) *IL-1 β* gene expression. The data are represented as mean ± SD, n = 8. Different letters indicate significant differences (*p* < 0.05).

The effects of TER on the cytokine content and gene expression in the colon in immune-stressed piglets are shown in Figure 3. The colonic contents of *IL-1β* and *TNF-α* in the LPS group were significantly greater than those in the CON group (*p* < 0.05), whereas the colonic contents of *IL-10* in the LPS group tended to decrease in comparison with those in the CON group (0.05 < *p* < 0.1). Compared with those in the LPS group, the PLT and PMT groups exhibited a substantial increase in *IL-10* (*p* < 0.05) and a significant decrease in *IL-1β* and *TNF-α* (*p* < 0.05) (Figure 3A–C). The gene expression of cytokines was also measured. Compared with those in the CON group, *TNF-α*, *IL-1β*, and *IL-18* gene expression in the LPS group significantly increased (*p* < 0.05). Compared with those in the LPS group, the relative expression levels of the *TNF-α*, *IL-1β*, and *IL-18* genes in the TER group were significantly lower (*p* < 0.05) (Figure 3D–F).

*NLRP3 expression in the colon*

**Figure 4:**
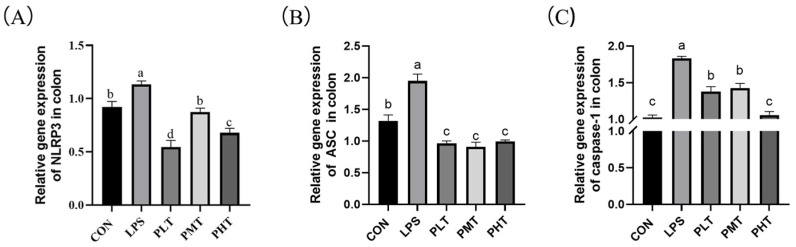
The effect of terpinen-4-ol on the expression of colitis-related genes in immune-stressed piglets. (**A**) *NLRP3* gene expression. (**B**) *ASC* gene expression. (**C**) *Caspase-1* gene expression. The data are represented as mean ± SD, n = 8. Different letters indicate significant differences (*p* < 0.05).

The effects of TER on the gene expression of the *NLRP3* inflammasome in the colon of immune-stressed piglets are shown in Figure 4. Compared with those in the CON group, the relative expression of the genes encoding *NLRP3*, *ASC*, and *caspase-1* in the LPS group increased (*p* < 0.05). Compared with those in the LPS group, the relative expression of the *NLRP3*, *ASC*, and *caspase-1* genes in the TER group decreased (*p* < 0.05) (Figure 4A–C).

*Alpha and beta diversity analysis*

**Figure 5:**
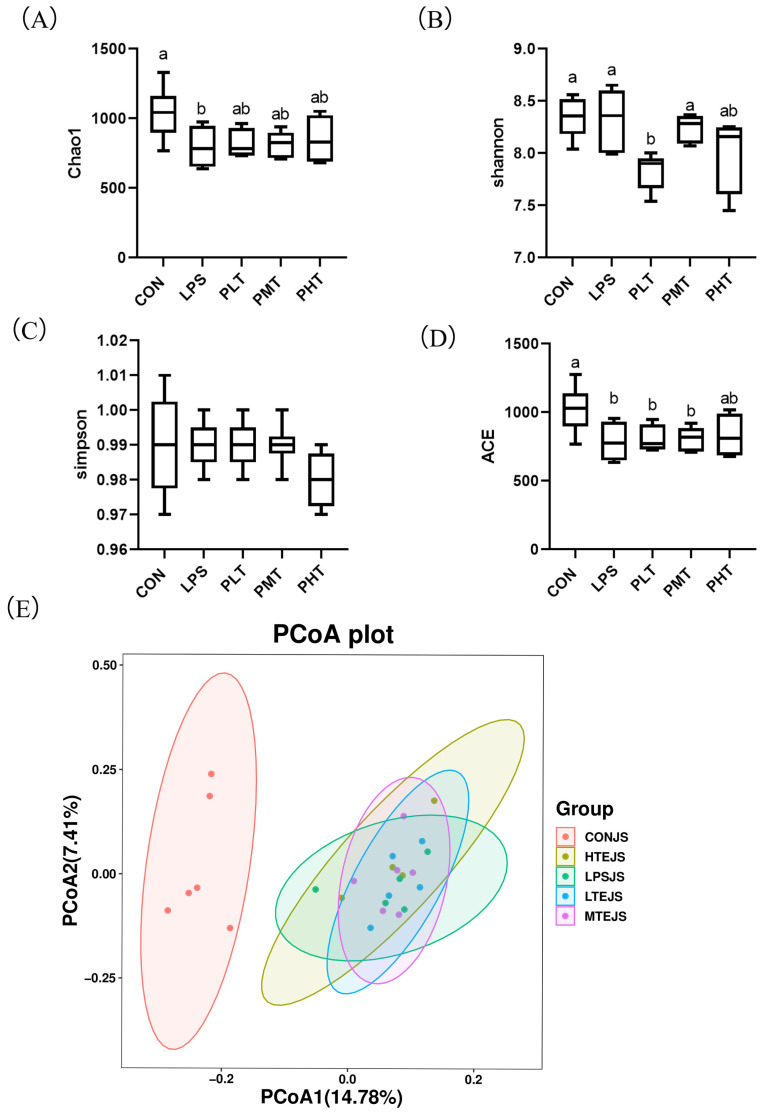
Terpinen-4-ol on the colon microbiota α and β. The impact of diversity analysis. (**A**) Chao1 index. (**B**) Shannon index. (**C**) Simpson index. (**D**) ACE index. (**E**) Principal component analysis. The data are represented as mean ± SD, n = 8. Different letters indicate significant differences (*p* < 0.05).

The effects of TER on the alpha and beta diversity of the gut microbiota of the colon in immune-stressed piglets are shown in Figure 5. The inclusion of TER led to an increase in the Chao1 and ACE indices compared with those in the LPS group; in contrast, the CON group showed a decrease in both indices. The Shannon and Simpson indices did not significantly differ across groups (*p* > 0.05) (Figure 5A–D).

*Bacterial abundance in the colonic mucosa*

**Figure 6:**
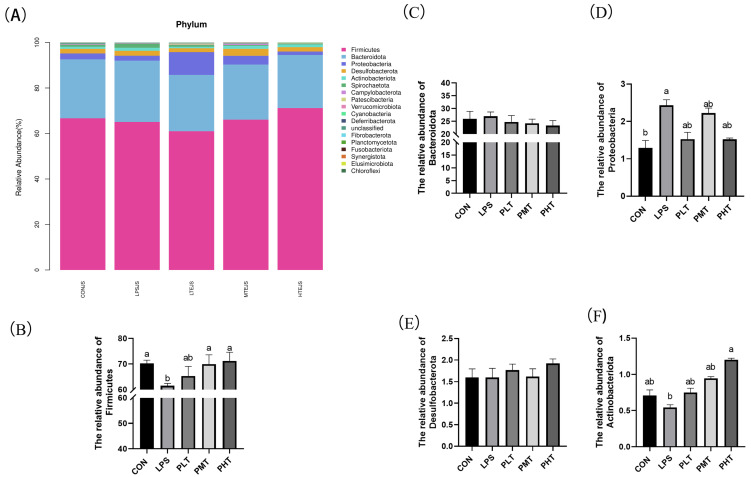
The effect of terpinen-4-ol on the gut microbiota of immune-stressed piglets. (**A**) Bacterial taxonomy analysis of gut microbiota at the phylum level. (**B**) Relative abundance of *Firmicutes*. (**C**) Relative abundance of *Bacteroidota*. (**D**) Relative abundance of *Proteobacteria*. (**E**) Relative abundance of *Desulfobacteriota*. (**F**) Relative abundance of *Actinobacteriota.* The data are represented as mean ± SD, n = 8. Different letters indicate significant differences (*p* < 0.05).

The effects of TER on the bacterial abundance in the colonic mucosa of immune-stressed piglets are shown in Figure 6. In terms of phyla, the three dominant phyla were *Firmicutes*, *Bacteroidetes*, and *Proteobacteria* (Figure 6A). The findings demonstrated that there was a substantial decrease in *Firmicute* abundance in the LPS group compared with that in the CON group (*p* < 0.05) (Figure 6B), and the abundance in all TER-treated groups was significantly greater than that in the LPS group. The abundance of the *Bacteroidetes* phylum tended to decrease with increasing doses of TER (0.05 < *p* < 0.1) (Figure 6C). There were no discernible differences in the abundances of the other bacteria between the groups (*p* > 0.05) (Figure 6D–F).

**Figure 7:**
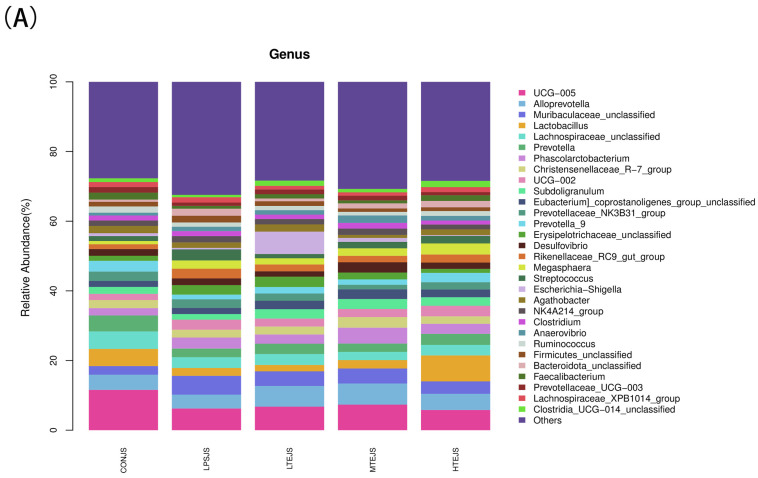

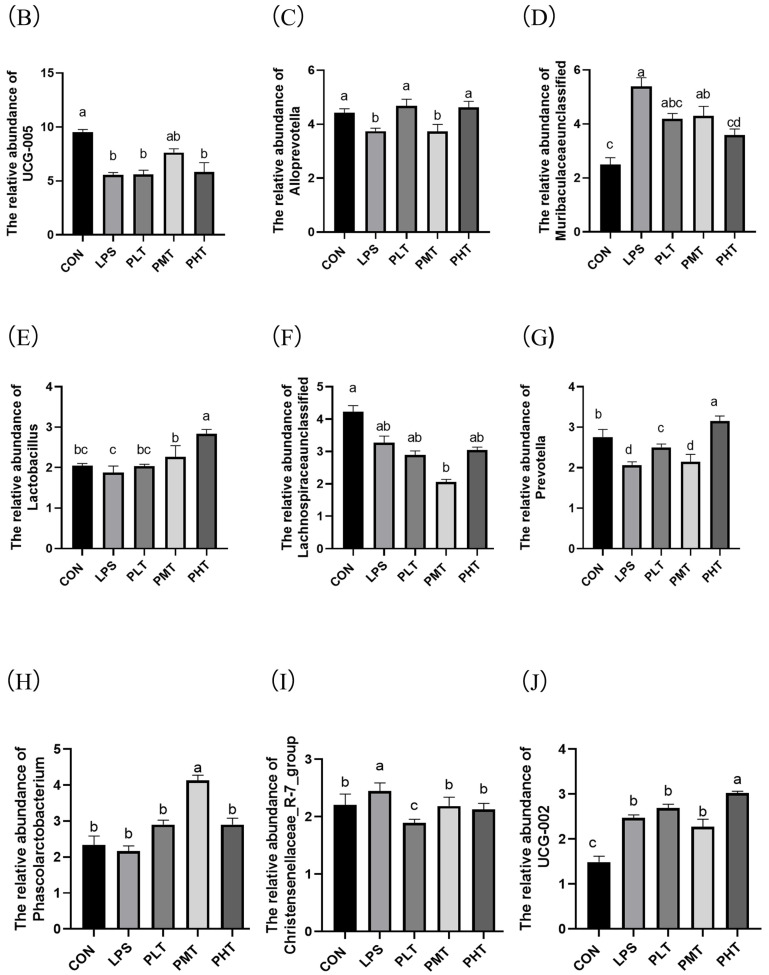
The effect of terpinen-4-ol on the gut microbiota of immune-stressed piglets. (**A**) Taxonomic analysis of gut microbiota at the genus level. (**B**) *UCG_ 005* relative abundance. (**C**) Relative abundance of *Alloprevotella*. (**D**) *Muribaculaceae* unclassified relative abundance. (**E**) *Lactobacillus* relative abundance. (**F**) *Lachnospiraceae* unclassified relative abundance. (**G**) *Prevotella* relative abundance. (**H**) *Phascolarium* relative abundance. (**I**) *Christensenaceae R-7_group* relative abundance. (**J**) *UCG-002* relative abundance. The data are represented as mean ± SD, n = 8. Different letters indicate significant differences (*p* < 0.05).

At the genus level, the colonic content included bacterial taxa such as *UCG-005*, *Paraprevotella*, *unclassified Bacteroidales*, *Lactobacillus*, *Christensenellaceae*, *Prevotella*, *Collinsella*, *Christensenellaceae*, and *UCG-002*, among others (Figure 7A). By comparing the LPS group to the CON group, the abundance of *UCG-005* was substantially lower (*p* < 0.05), whereas in the PMT group, it was significantly greater (*p* < 0.05) (Figure 7B). Compared with the CON group, the abundance of *Muribaculaceae_unclassified* in the LPS group was significantly elevated, while that in the PHT group was significantly reduced (*p* < 0.05) (Figure 7D). Compared with the CON group, the abundance of *Prevotella* in the LPS group was significantly decreased (*p* < 0.05) (Figure 7G). Compared with the CON group, the abundance of *Christensenellaceae_R-7_group* in the LPS group was significantly increased, while that in the TER group was significantly decreased (*p* < 0.05) (Figure 7I). Compared with the CON group, the abundance of *UCG-002* in the LPS group was significantly enhanced (*p* < 0.05) (Figure 7J).

## 4. Discussion

Intestinal health in piglets has always been a primary concern in the livestock production industry. Immune stress leads to intestinal inflammation and damage and causes diarrhea and growth inhibition, which in turn severely harms the health of piglets and reduces the economic benefits of breeding [23,24]. Our earlier research showed that TER may successfully reduce small intestine intestinal inflammation, which can improve the growth performance of weaned piglets. However, the effects of LPS on the intestinal barrier function of the colon have not been investigated, and the regulatory effects of TER on the barrier function of the colon in piglets remain unclear. Hence, the regulatory effects of TER on intestinal barrier function in the colon of immune-stressed weaned piglets were investigated in this study. The findings of this investigation might offer fresh evidence in favor of TER use.

The rate of intestinal epithelial cell renewal is indicated by the depth of the crypt, with undifferentiated cells continuously generated in crypts, migrating to the villi tips to mature, and replacing shedding cells [25]. In this study, LPS stimulation resulted in a decrease in crypt depth, while TER supplementation increased crypt depth in the colon of LPS-stimulated piglets. Our results were in accordance with previous studies on DSS stimulation model in mice. DSS may also damage the integrity of the intestinal mucosal barrier, thus causing the invasion of the components of the bacteria including LPS, thus indirectly triggering systemic inflammatory responses similar to those caused by LPS [5]. A previous study showed that DSS stimulation could also cause a decrease in crypt depth in the colon, thus inducing colitis in mice [26]. *Berbonetia papyrifera* leaf extract (containing terpenoids) alleviated the reduction in colonic crypt depth in DSS-treated mice [27]. The findings of the present study suggest that TER might alleviate the impairment of intestinal barrier function induced by LPS through the regulation of intestinal epithelial cell regeneration in the colon of weaned piglets. Healthy colonic crypts can also enhance the absorption efficiency of nutrients and strengthen the immune system function, reducing the incidence of diseases, thereby promoting rapid growth and development in piglets [28]. The increased crypt depth in the colon of piglets may also explain the improved growth performance in the 60 mg/kg TER supplementation group to some extent.

Goblet cells, which are prevalent in the colon epithelium, secrete mucin, which is essential for intestinal epithelial function [29]. Muc2 is the primary mucin produced by intestinal goblet cells and serves as a barrier between colonic epithelial cells and bacteria [30,31,32]. Decreased MUC2 expression, as observed in some bacterial infections, can compromise mucosal integrity [33]. Weaning stress also impairs the intestinal physical barrier and reduces the number of goblet cells in the colon [34]. In this study, the number of goblet cells and the expression of MUC2 were decreased in the LPS group, indicating that the integrity of the intestinal mucosa was disrupted after LPS stimulation. Additionally, previous studies have shown that mice given DSS have fewer goblet cells, but animals with DSS-induced colitis have significantly more goblet cells when administered appropriate doses of TER [14]. Previous studies have indicated that the administration of polyphenol extract (containing terpenoids) from jujube to mice in the DSS group can significantly mitigate the decrease in MUC2 expression [35]. Our results are consistent with those of previous studies. Previous studies suggest that an appropriate dose of TER has a protective effect on the colon; however, high doses of TER appear to have a detrimental impact on intestinal health [14]. In this study, high doses of TER supplementation caused a decrease in goblet cells and MUC2 in colon compared with that in the medium dose TER-supplemented group. The reasons might be associated with TER at high concentrations having certain toxic effects on the intestinal epithelial cells, including goblet cells. The results highlight the importance of dose optimization when administering TER. And the results are in accordance with our previous in vitro study [36]. In addition, improved goblet cell function and MUC2 expression can enhance intestinal health and nutrient absorption, thereby promoting piglet growth and development [37], which may also explain the improved growth performance (data not published) in the 60 mg/kg TER supplementation group.

The antioxidant function of the body is usually reflected through indicators such as *SOD*, *GSH-Px*, MDA, *CAT*, and T-AOC. An increase in the levels of *SOD*, *GSH-Px*, *CAT*, and T-AOC, along with a decrease in the MDA level, indicates an increase in the antioxidant capacity of the body [38]. In rats with ulcerative colitis, the colonic MDA level is elevated, while the levels of *GSH-Px* and *SOD* are reduced [39]. Studies have also shown that LPS stimulation leads to decreased T-AOC, *CAT*, and *SOD* activities and increased MDA content in the jejunum of piglets [40]. The results of this study also show that LPS stimulation decreases the T-AOC, *CAT*, and *SOD* activities and increases the MDA content in the colon of piglets. The results of this study indicate that LPS stimulation causes an imbalance in the oxidative equilibrium in the colon of piglets. In this study, we also observed a dose-dependent biphasic effect of TER on oxidative stress markers. At low concentrations, TER exhibited antioxidant properties, reducing MDA activity by neutralizing reactive oxygen species (ROS) and enhancing antioxidant enzyme activities such as *SOD*, thereby decreasing cellular oxidative stress [41]. However, at higher concentrations, TER induced oxidative stress, increasing MDA activity. This suggests that at high doses, excessive ROS production can overwhelm antioxidant defenses, leading to decreased *SOD* activity and promoting lipid peroxidation [42]. These findings align with previous studies, indicating that proper doses of TER can be protective, while high doses may cause cellular toxicity and oxidative damage, a common pattern for various chemicals and drugs. In most cases, antioxidant enzyme activity aligns with gene expression. However, the discrepancy between gene expression and activity of antioxidant enzymes arose because antioxidant enzyme activity is influenced not only by gene expression but also by post-translational modifications, enzyme stability, and cellular redox state regulation. Even with increased gene expression, the post-translational modifications or its binding affinity to substrates may vary, resulting in activity changes that do not entirely correspond with gene expression [43]. In the present study, 60 mg/kg of TER supplementation reduced the content of MDA and increased the levels of *GSH-Px*, *CAT*, and *SOD* in the colon of immune-stressed piglets. Our earlier research also showed that adding tea tree oil (or TER, the primary functional component) to the ileum of pigs increased *GSH-Px* and *SOD* activity and decreased MDA levels [44]. The results of this study suggest that an appropriate dose of TER scavenges free radicals and enhances the antioxidant capacity, thus improving the chemical barrier function of the colon in immune-stressed piglets.

Numerous studies have emphasized the important role of NLRP3 in intestinal barrier function [45]. When cells in the body are stimulated by exogenous microorganisms such as viruses and bacteria, the *NLRP3* protein recruits *ASC* and *pro-caspase-1* to form the *NLRP3* inflammasome [46]. *NLRP3* then activates *caspase-1*, thereby promoting the maturation and secretion of the inflammatory cytokines *IL-1β* and *IL-18* [47]. A previous study reported that LPS can induce the activation of *NLRP3* and upregulate the expression of *NLRP3* and *pro-caspase-1* in mice [48]. Previous studies also showed that LPS induced increased secretion of proinflammatory cytokines, including *TNF-α* and *IL-1β*, in the jejunum of weaned piglets [49]. Previous studies have also suggested that the gene expression of *IL-1β* and *IL-18* is significantly increased after an intraperitoneal injection of LPS in the jejunum of mice [50]. In this study, *IL-1β* gene expression decreased with PLT and PMT, but increased with PHT. Our previous researches showed that a high concentration of tea tree oil (the main functional component is TER) increased gene expression of *IL1β* in IPI-2I cells, which was consistent with the results in this study [51]. In this study, *IL-10* levels increased with low and moderate doses of TER, but decreased with high doses. We speculated that at low and moderate doses of TER, the immune system’s anti-inflammatory response was activated, leading to an increase in *IL-10* levels. At high doses, TER might exhibit certain cytotoxicity, directly or indirectly impairing immune cell function, which resulted in the reduced synthesis of *IL-10* and other cytokines, thereby suppressing the immune response [52]. The results of this study showed that LPS increased the secretion of proinflammatory cytokines and upregulated the expression of proteins in the *NLRP3* inflammasome in the colon, which is in accordance with previous studies. These findings suggest that LPS may trigger inflammation by activating the *NLRP3* inflammasome. Additionally, prior research has shown that TER dose-dependently reduces the expression of *NLRP3*, *ASC*, and *caspase-1* in the colon of mice after DSS stimulation [53]. The results of this study show that TER inhibits the production of *NLRP3*, *ASC*, and *caspase-1* and decreases the secretion of proinflammatory cytokines in the colon of LPS-stimulated piglets, which suggests that TER might alleviate LPS-induced intestinal inflammatory injury through the inhibition of inflammasome pathways, thus improving the immune barrier function in the colon of piglets.

Plant-derived extracts can improve gut health by modulating the composition of the microbiome [54]. TER exhibits broad-spectrum antibacterial and anti-inflammatory effects, which are crucial for maintaining intestinal homeostasis [55]. In this study, LPS-induced dysbiosis was observed in the colon of piglets, characterized by an increase in the abundance of *Proteobacteria* and *Bacteroidota*, and a decrease in *Firmicutes*. These microbial changes are commonly associated with inflammation and impaired gut health [56]. However, supplementation with TER restored a more balanced microbial composition by increasing the abundance of *Firmicutes* and beneficial bacteria such as *Phascolarctobacterium*, *Alloprevotella*, and *UCG-005*, while decreasing the abundance of potentially harmful taxa like *Bacteroidetes* and *Proteobacteria*. The abundance of *Proteobacteria* and *Bacteroidetes* increased and the abundance of *Firmicutes* decreased in the colon of DSS-stimulated mice, while *Scutellaria baicalensis* Georgi polysaccharide supplementation restored the dysregulated microbiota to normal levels [57]. The modulation of the gut microbiome by TER can be explained by its antimicrobial properties, which likely contribute to reducing the levels of harmful bacteria and promoting the growth of beneficial species. At the genus level, studies have shown that the relative abundance of *Alloprevotella* was decreased in LPS-stimulated piglets [58]. Additionally, plant-derived extracts, such as chito-oligosaccharides, have been found to significantly increase the relative abundance of beneficial bacteria such as *Phascolarctobacterium*, *Prevotella*, and *Prevotella_9* in the gut of piglets, thereby reducing disease risk [59]. Furthermore, the increased abundance of *Phascolarctobacterium* has been linked to enhanced antioxidant capacity [60] and a negative correlation with proinflammatory cytokines such as *IL-1β* [61]. The research findings indicate that LPS significantly reduces the abundance of *UCG-005* and *Prevotella*. This suggests that LPS disturbs the intestinal microecology and inhibits the growth of beneficial bacteria when inducing intestinal inflammatory responses. However, the addition of an appropriate dose of TER is observed to increase the abundance of *UCG-005* and *Prevotella*. Previsou studies have shown that bacteria such as *Phascolarctobacterium* and *Alloprevotella* play roles in maintaining gut health and regulating immune responses [62]. By increasing the abundance of beneficial bacteria including *Alloprevotella*, *Prevotella*, and *Phascolarctobacterium*, TER may alleviate inflammation and oxidative stress in the gut, thereby strengthening the intestinal barrier function. Through its impact on microbial diversity and composition, TER can help reduce intestinal permeability and protect against inflammation-induced gut damage, which is commonly observed in conditions such as IBD and stress-induced colitis [53]. The results of this suggest that TER supplementation may also alleviate IBD and colitis in human beings, and further studies are needed. By modulating microbial populations and enhancing beneficial bacteria, TER may help restore a balanced gut microbiome, optimize intestinal barrier function, reduce inflammation, and ultimately improve overall gut health.

## 5. Conclusions

In summary, proper doses of TER supplementation increased the depth of the colonic crypts, the number of goblet cells, and MUC2 expression, decreased proinflammatory cytokine secretion, downregulated the expression of genes in the *NLRP3* inflammasome, reduced the proportions of *Bacteroidetes* and *Proteobacteria*, and increased the proportions of *Firmicutes*, *Phascolarctobacterium*, *Alloprevotella*, and *UCG-005*, thus improving the intestinal barrier function of the colon in LPS-stimulated piglets. An amount of 60 mg/kg TER supplemented in the diets of weaned piglets is recommended based on the results of this study.

## Figures and Tables

**Table 1:** Composition and nutrient levels of the basal diet.

Ingredient	Content
Raw materials, %	
Corn	10.00
Fish meal Puffed corn Soybean oil Fermented soybean meal Soybean preconcentrated protein Whole soybeans Whey powder Flour Rice husk powder Glucose	5.83 27.67 1.67 8.00 2.50 6.50 15.00 10.00 0.33 2.50
Carrier rice husk powder	2.31
Calcium hydrogen phosphate Lysine	0.15 0.43
Methionine	0.30
Choline chloride Salt Premix ^(1)^ Total 100.00	0.12 0.30 1.39 100.00
Nutritional levels ^(2)^	
Digestible energy (kcal/kg)	3.33
Crude protein, %	18.83
Calcium, %	0.43
Total phosphorus, %	0.52
Lysine, %	1.44
Methionine, %	0.62
Threonine, %	1.00
Tryptophan, %	0.35

^(1)^ The premix provided the following per kg of the diet: Fe 53 mg, Gu 5 mg, Mn 13 mg, Zn 40 mg, Co 0.06 mg, Se 0.23 mg, I 0.33 mg, VA 13 500 IU, VD_3_ 2 750 IU, VE 6.25, VK_3_ 1.25 mg, thiamine 0.5 mg, riboflavin 3.75 mg, pantothenic acid 6.25 mg, nicotinic acid 8.75 mg, adermin 0.5 mg, VB_12_ 0.01 mg, biotin 0.013 mg, folic acid 0.125 mg, phytase 500 mg, sweetener 200 mg, sodium glutamate 1000 mg, mold adsorbent 500 mg, antioxidant 200 mg. ^(2)^ DE was a calculated value, while the other nutrient levels were measured values.

**Table 2:** Primers used in this study.

Genes	Accession No.	Primer Sequence (5′ to 3′)
*β-Actin*	DQ845171.1	F AGGCCAACCGTGAGAAGATG
		R CATGACAATGCCAGTGGTGC
*CAT*	NM 214301.2	F CTGTAAGGCTAGTCGGACACC
		R ATATCAGGTTTCTGCGCGGC
*SOD1*	NM 001190422.1	F GTGCAGGGCACCATCTACTTC
		R GATCACCTTCAGCCAGTCCTT
*Gpx1*	NM 001206359.1	F CTAGCAGTGCCTAGAGTGCC
		R CGCCCATCTCAGGGGATTTT
*NLRP3*	NM001256770.2	F TGTATTGAGAACTGTCGCCATGTGG
		R CTCCTCTTCCTCCTCCTCCTCTTTG
*ASC*	XM003124468.5	F GAAGGTGCTGACGGAAGAGC
		R TCCTTGCAGGTCAGGTTCCA
*Caspase-1*	NM214162.1	F CCAGTTAAGCCTGCGTCTTCAGAG
		R GGCGTGTGCGAATTGATTTTCCC
*IL-1β*	NM001302388.2	F AAGAGGGACATGGAGAAGCGATTTG
		R TTGTTCTGCTTGAGAGGTGCTGATG
*IL-18*	NM213997.1	F AGACCTGGAATCGGATTACTTTGGC
		R ACGGCTTGATGTCCCTGGTTAATG
*TNF-α*	NM214022	F CCACCACGCTCTTCTGCCTAC
		R TTGAGACGATGATCTGAGTCCTTGG

## Data Availability

The original contributions presented in this study are included in this article; further inquiries can be directed to the corresponding authors.
